# Image Classification in JPEG Compression Domain for Malaria Infection Detection

**DOI:** 10.3390/jimaging8050129

**Published:** 2022-05-03

**Authors:** Yuhang Dong, W. David Pan

**Affiliations:** Department of Electrical and Computer Engineering, University of Alabama in Huntsville, 301 Sparkman Dr NW, Huntsville, AL 35899, USA; yd0009@uah.edu

**Keywords:** compression domain, JPEG, classification, malaria

## Abstract

Digital images are usually stored in compressed format. However, image classification typically takes decompressed images as inputs rather than compressed images. Therefore, performing image classification directly in the compression domain will eliminate the need for decompression, thus increasing efficiency and decreasing costs. However, there has been very sparse work on image classification in the compression domain. In this paper, we studied the feasibility of classifying images in their JPEG compression domain. We analyzed the underlying mechanisms of JPEG as an example and conducted classification on data from different stages during the compression. The images we used were malaria-infected red blood cells and normal cells. The training data include multiple combinations of DCT coefficients, DC values in both decimal and binary forms, the “scan” segment in both binary and decimal form, and the variable length of the entire bitstream. The result shows that LSTM can successfully classify the image in its compressed form, with accuracies around 80%. If using only coded DC values, we can achieve accuracies higher than 90%. This indicates that images from different classes can still be well separated in their JPEG compressed format. Our simulations demonstrate that the proposed compression domain-processing method can reduce the input data, and eliminate the image decompression step, thereby achieving significant savings on memory and computation time.

## 1. Introduction

Owing to the rapid improvement of GPU computing power, neural networks are now able to grow into larger sizes in both width and depth. However, more layers bring an increasing number of parameters that need to be trained. For example, GoogLeNet [1] has 6 million parameters, AlexNet [2] has 62 million parameters, and VGG16 [3] even has 138 million parameters. Researchers have been studying how to reduce the parameter size of neural networks and have successfully used different pruning methods to relieve the pressure of GPUs [4]. In this paper, instead of fixating on the neural network, we investigated the feasibility of using the neural network to classify images in their compressed domain, which took much less storage and thus consumed less computing power.

We evaluated different combinations of output from the JPEG lossy compression method. We calculated the DC values of each minimum coded unit (MCU), together with zero to five AC values after 2D discrete cosine transform (DCT) or quantization. We defined these datasets as the baseline because all the DCT coefficients were directly correlated to the pixel values from the original image. DC values after differential pulse-code modulation (DPCM) and variable length coding (VLC) were also extracted.

For the compressed bitstream, two datasets were built from the scan segment in both decimal and binary numbers. Finally, the entire bitstream was represented in decimal and binary values. All the mentioned datasets were tested on a long short-term memory (LSTM) network [5].

The rest of this paper will be organized as follows. In Section 2, we will introduce the research that had been conducted in the compression domain. Section 3 will cover the source image we used, a simple illustration of how JPEG compressed an image, and how we extracted data from different stages during compression. Section 4 contains the classification results and analysis. Finally, Section 5 concludes this paper.

## 2. Related Work

Research has been conducted on evaluating how the JPEG compressed image would affect classification accuracies for deep learning [6,7], including one of our previous publications [8]. The input samples were images with different compression qualities. However, true compression domain data were generally difficult to work with, because the transformation, prediction, and other non-linear operations inside the compressor together contributes to a less correlated bitstream.

A comprehensive description of text analytics directly on compression was provided in [9,10], but the analysis was limited to saving in storage and memory. Bits after compression of the text file were also used in [11] to distinguish 16 dictionary-based compression types. Although different methods were proposed in [12,13], the core concept was the same, which was to realize random data access in compressed bitstream. However, for JPEG compression with sequential mode, extracting DCT coefficients requires full decompression. This concept might find new applications in other image compression formats.

Other researchers computed the normalized compression distance from the length of compressed data files for classification [14,15,16]. Though this method worked in various areas, the compression had to be lossless. The keyframe of compressed video data was also evaluated in [17,18,19,20]. The compressed domain data of High-Efficiency Video Coding was also used for object detection in [21,22,23,24]. However, only special sections based on the coding syntax were decoded.

Some studies [25] used DCT to generate coefficients for classification. However, the DCT was conducted on whole images, instead of 8×8 minimum coded (MCU) unit in JPEG. DCT coefficients were also used for image retargeting [26], image retrieval [27,28] and image classification [29]. Compressed bitstream was used in [30] to classify images. As the input images were sequentially encoded, it is also required to decode the whole bitstream first to get DC and AC values for each MCUs.

## 3. Materials and Methods

### 3.1. Source Image

**Figure 1:**
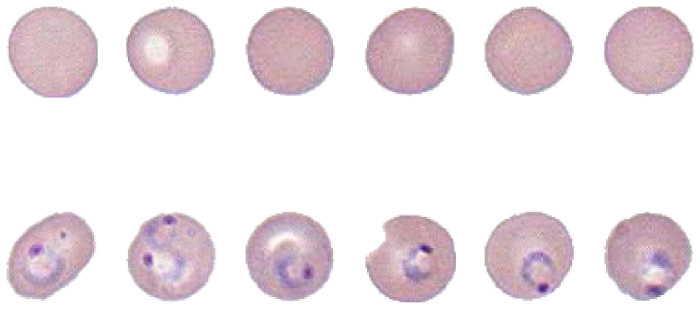
Cropped red blood-cell samples. Top row shows non-infected cells, bottom row shows infected cells. The purple nucleus and blue ring make the malaria parasite.

We used whole slide images (WSI) from the University of Alabama in Birmingham’s pathology lab [31]. The entire image contained around 1,000,000 red blood cells, with at least 0.2% samples infected by the malaria parasites. We performed several image morphological operations to crop each cell out [32]. Then, we used the support vector machine (SVM) to classify cells based on several selected features [33]. The classified data were provided to pathologists for curation. Finally, we compiled the pure malaria-infected-cell dataset and the normal red blood-cell dataset as shown in Figure 1.

The data sizes for training, testing, and validation are listed in Table 1, with the ratio of sample numbers regarding the whole input set equating to roughly 0.70:0.15:0.15. Each cell image was resized to 50×50 for the unified dimension and transformed into a grayscale image for simplicity.

### 3.2. Discrete Cosine Transform

Discrete cosine transform uses the summation of cosine functions at different frequencies and magnitudes to represent the original input. It is widely used in signal processing and data-compression schemes because the input signal energy can be concentrated in just a few coefficients after the transformation. For an 8×8 MCU in JPEG compression, we used Equations (1) and (2) to calculate DC and AC values after DCT.

(1) $$DC=\frac{1}{8}\sum_{m=0}^{7}\sum_{n=0}^{7}S_{mn},$$

(2) $$AC_{xy}=\frac{1}{4}\sum_{m=0}^{7}\sum_{n=0}^{7}S_{mn}cos\frac{\left(2m+1\right)x\pi }{16}cos\frac{\left(2n+1\right)y\pi }{16}.$$

**Figure 2:**
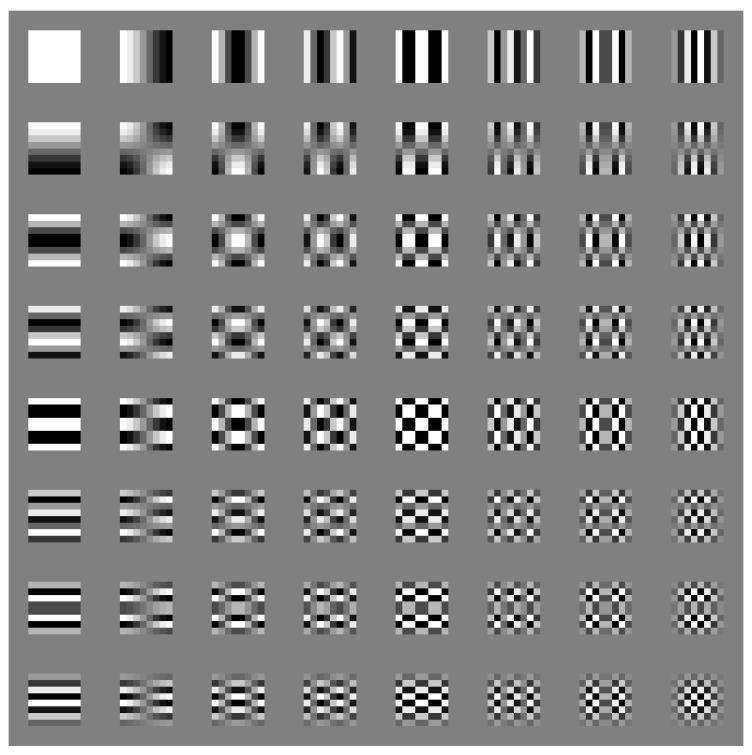
Basis matrices of DCT in JPEG compression. The block on the top left is the DC component. The frequency increases with the increment of both row and column numbers.

We can calculate basis matrices of DCT using the above equations and visualize them in Figure 2.

DCT is related to discrete Fourier Transform (DFT). In fact, for a sequence of length *N*, DFT assumes the sequences outside are replicas of the same sequence, which will introduce discontinuities at both ends of the sequence. Meanwhile, for DCT, the sequence will be mirrored to make length 2N. The DCT is simply the first *N* points after a 2N-length DFT. The mirroring operation will eliminate the discontinuities at both ends of the sequence, which means that if we discard the high-frequency component, the remaining coefficients will not create additional distortion. Additionally, the result after DCT being real values makes it more convenient to use in real applications.

### 3.3. JPEG Flow Chart

**Figure 3:**
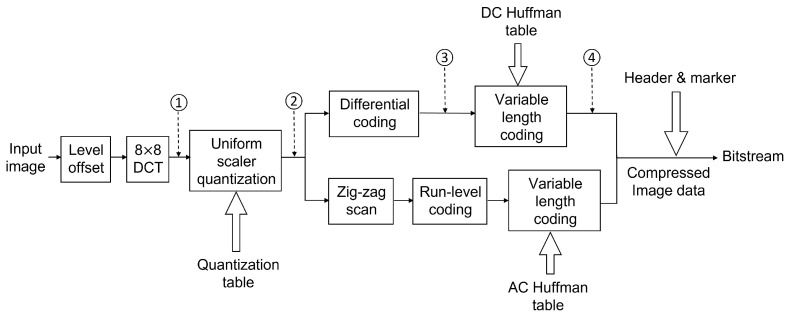
Flow chart for baseline JPEG for the grayscale image. An input image will go through multiple operations, including DCT, quantization, differential coding, and variable-length coding. The output is bitstream with headers and markers containing side information for the decoder. Numbers 1 to 4 denote the locations where we extract datasets 1 to 4.

The first step of baseline JPEG compression was to perform a level shift. Since our input images had a bit depth of eight, every pixel value was subtracted by $2^{8}=128$ to reduce the dynamic range. Then, each image was divided into multiple MCUs with the size of 8×8. After applying DCT, for each MCU, we calculated one DC coefficient using Equation (1) and 63 AC coefficients using Equation (2). The flow chart of baseline JPEG compression is shown in Figure 3.

Then, the 64 DCT coefficients were divided by a quantization matrix and rounded to the nearest integers. The DC and AC values were separated from here for different coding schemes. All of the quantized DC values were gathered and transferred using DPCM, and VLC was applied with the DC Huffman code table. The remaining 63 AC values were first reordered using a zig-zag scan pattern, which could be found in Appendix A. Then the 63-point vector was run-length coded, and then mapped to a binary stream using the AC Huffman table.

The two bitstreams for the DC components and AC components were reorganized with markers and headers, which contained side information, including the size of the image, length of the current segment, and the location of the target Huffman code table during compression or decompression. Since we ran codes in the MATLAB environment, we used the same quantization matrix, DC Huffman table, and AC Huffman table as MATLAB builtin function *imwrite* for JPEG compression. See the three tables in Appendix A.

### 3.4. Extract Coefficients

We wrote a simple version of MATLAB code based on the JPEG standard [34], which could only compress grayscale images to analyze how data from different stages during compression would impact classification. Then, we could easily modify the program to produce the data we need.

The first approach was to take the DC coefficients, combined with zero to five AC coefficients directly after DCT, then make six datasets as denoted by the circled number one in Figure 3. We also generated another six datasets from the DCT coefficients after quantization using the same strategy, as the circled number two in the same figure. Using the DC value with several AC values to represent the whole MCU was reasonable because most energy was concentrated on the left top corner in the DCT coefficient matrix, which was the low-frequency region as shown in Figure 2. In addition, the large values on the right bottom corner of the quantization table further reduced the impact of the high-frequency component. See Table 2 for an example of how we built these 12 datasets from a sample MCU after a level shift in Table 3.

We also extracted DC values after DPCM and VLC to evaluate how these two operations would impact the classification accuracy. These two approaches are marked as circled numbers three and four in Figure 3. Note that after DPCM, the coded DC values were still integers. However, after VLC, they were all turned into bits according to the DC Huffman table.

**Figure 4:**
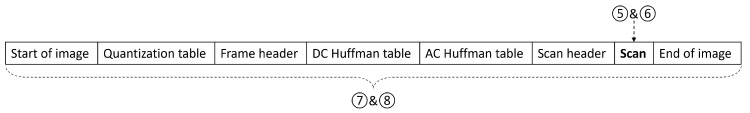
Simple syntax of JPEG compression. Each block is represented in hexadecimal values. Numbers 5 to 8 are locations where we extract datasets 5 to 8.

For the compression domain, we first selected the “scan” segment, which contained decimal values after compression without most headers and markers. This method was marked as circled number five in Figure 4. We also extracted the corresponding binary form of the “scan” segment as dataset number six. Finally, we tested the whole compressed image data in both binary form and decimal form, as circled numbers seven and eight in Figure 4.

For datasets five to eight, as the coded length of each image would differ from each other, we had to specify the length of the input as *D*. Any input with a size larger than *D* was truncated, and the ones that were shorter than *D* were padded with zero to make the same lengths. To evaluate how the classification accuracies would react to the change of input length, we also selected 10 datasets with linearly increased *D* as input to the LSTM. Descriptions of all eight datasets are listed in Table 4 below.

## 4. Results and Analysis

**Figure 5:**
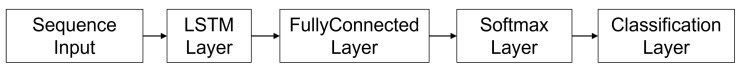
LSTM model for training, validation, and testing. Input is datasets one to eight, and the final classification layer gives the result of whether the input sample is infected or not.

The above eight datasets were fed into LSTM for classification. The structure of the model we used is shown in Figure 5. We chose the neuron numbers to be 250, and the training epoch to be 30. The reason for choosing LSTM over CNN was that the input data was a bitstream (or bytestream if decimal values were used). After compression, the coded bitstream lost the original correlation existing in neighbor pixels, when the image was divided into MCUs. During the compression procedure, the most important reason for CNNs, the spatial correlation, was largely reduced, making CNN less efficient than LSTM, which worked well for sequential input.

### 4.1. Coefficients after DCT and after Quantization

**Figure 6:**
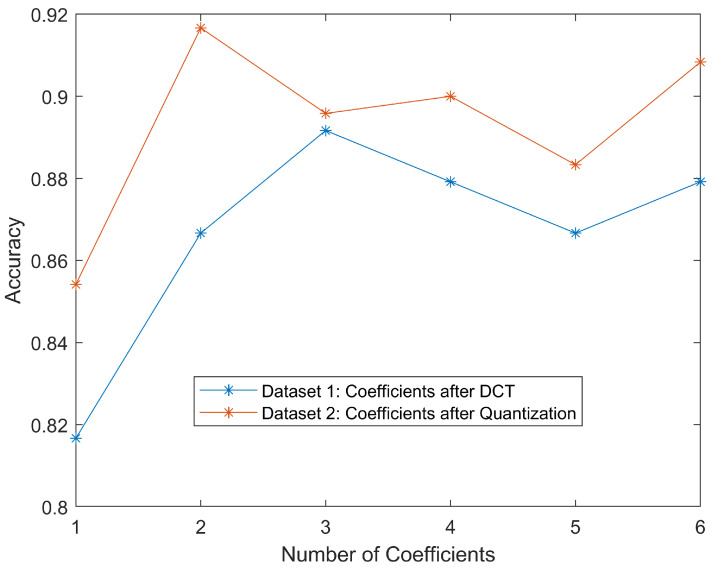
Classification accuracies on coefficients after DCT and after quantization. Generally, an increasing number of DCT coefficients leads to higher accuracies.

For datasets one and two, we combined the curves for each set in Figure 6 to show how classification accuracies would change regarding DC values with different numbers of AC values. Generally, the classification was more accurate as more DCT coefficients were involved. More coefficients could be interpreted as more information came from the original image, making classification easier. It is worth noting that even using only DC value, the accuracy could still reach greater than 80%. This should be credited to the simplicity of the dataset. The DC value of the MCU containing the malaria parasite would be much larger than that of red blood cell cytoplasm because the parasite was stained purple/blue. Therefore, DC values were one of the most important features. We could also notice that for these two datasets, the classification accuracies of coefficients after quantization seemed higher than the ones after DCT. This could be attributed to the quantization reducing the dynamic range of the coefficients.

As we can see, all accuracies were above 80%, meaning we could use the DC values alone, or DC value adding a few AC values, to achieve a reasonably good performance. However, due to the sequential decoding mechanism of JPEG decompression, we still had to decode the whole bitstream to get these values.

### 4.2. DC Values after DPCM and VLC

**Figure 7:**
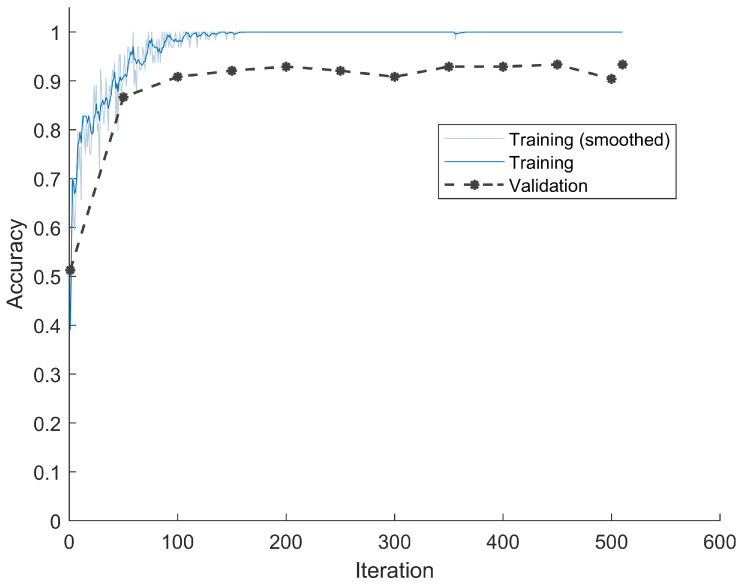
Classification accuracies and loss during training and validation for DC values after DPCM. The accuracy curve shows that even using only DC value after DPCM, the LSTM model converges well after certain iterations.

Dataset three contained only DC values after DPCM. The classification accuracy versus training iteration is shown in Figure 7. Due to the fact that DPCM actually subtracted the previous DC value from the current one, the redundancy was further reduced, thus pushing the accuracy even higher. The classification accuracy on the validation set was 92.92%, and on the testing set was 94.17%.

**Figure 8:**
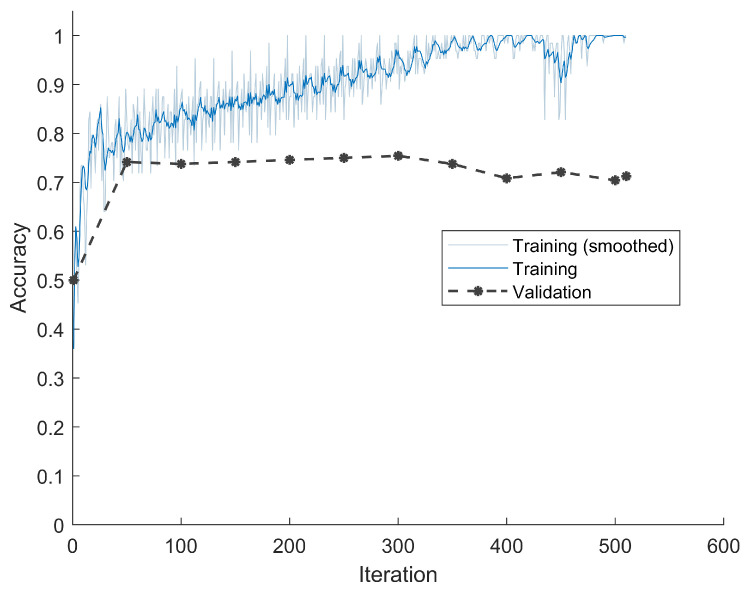
Classification accuracies and loss during training and validation for DC values after VLC. The average performance denotes that VLC devastated the correlation between pixels and bitstream.

We also calculated dataset four, which consisted of bits corresponding to DC values after VLC. The classification accuracy was lower than the previous result, as shown in Figure 8, and the result on the testing set was 69.58%. The low value was because the bitstream was naturally decorrelated, and furthermore, different coding mechanisms were used for positive and negative values after DCT, which further reduced the correlation between bitstream and pixel values. Additionally, it was hard for LSTM to generalize the input data, because the neural network had overfitting after 10 epochs. We also had to choose the model with minimum validation loss.

### 4.3. Scan Segment in Decimal and Binary

**Figure 9:**
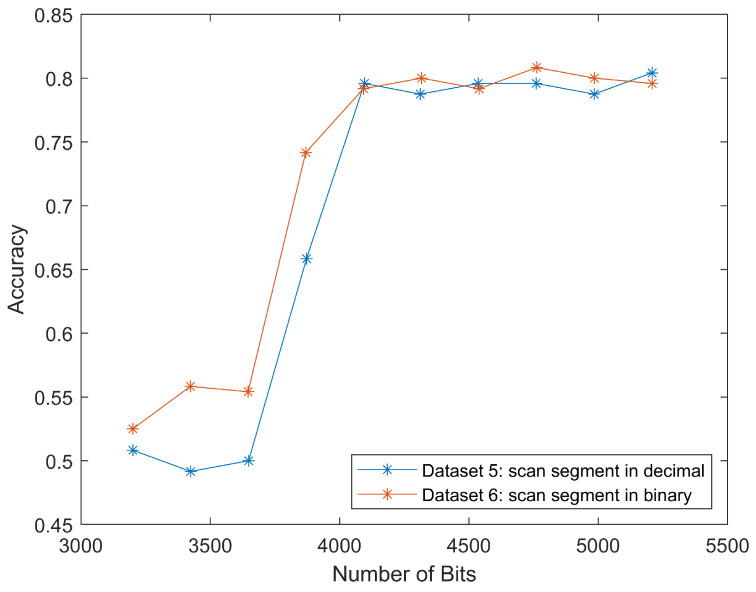
Classification accuracies for different lengths of scan segment in decimal or binary form. The sharp increase and good match of the two curves prove the scan segment can be classified in both decimal and binary form.

Datasets five and six were scan segments from the compression domain in decimal and binary form, respectively. Because the original *x* axis for dataset five was “number of bytes”, we multiplied the input length vector by eight to get the number of bits and resulted in a better comparison, as shown in Figure 9. Since the accuracies for inputs with bits number smaller than 3600 kept oscillating around 50%, which was equal to the result of flipping a coin, we omitted most data points in this range for better visualization. In the same figure, there is a sharp increase between 3600 to 4000 bits. After that, the accuracy remains around 80%. This could be attributed to the location of important features for classification in this special case. For malaria-infected red blood cells such as those in Figure 1, the most useful features—the purple nucleus and the blue ring shape of the parasite—often presented in the middle region of the image. Similarly, the corresponding bits for these features also started to appear around 3600 bits to 4000 bits, thus increasing the classification accuracy.

### 4.4. Whole String in Decimal and Binary

**Figure 10:**
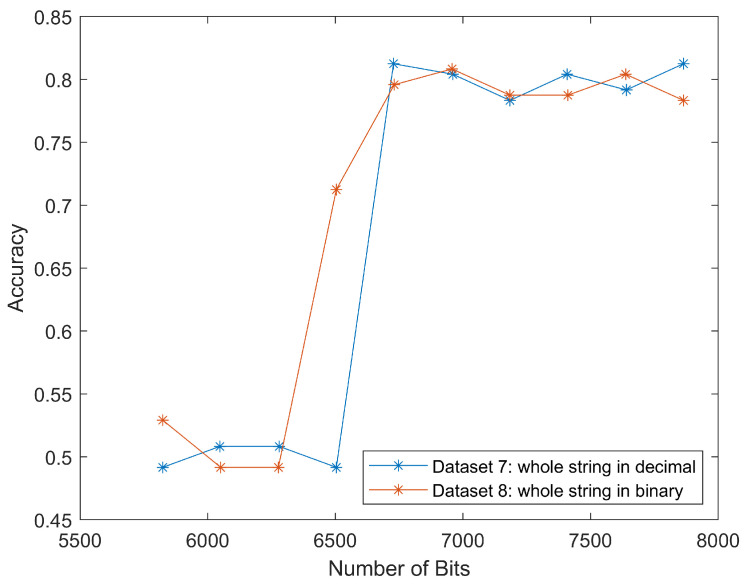
Classification accuracies for different length of whole string in decimal or binary form. The tread of two curves proves the whole string in both decimal and binary form can be classified.

The results for datasets seven and eight were similar to that of datasets five and six, as shown in Figure 10. The classification accuracy rapidly increased to 80% around 6500 bits. This meant that we could classify the scan segment alone and achieve a similar classification performance.

**Figure 11:**
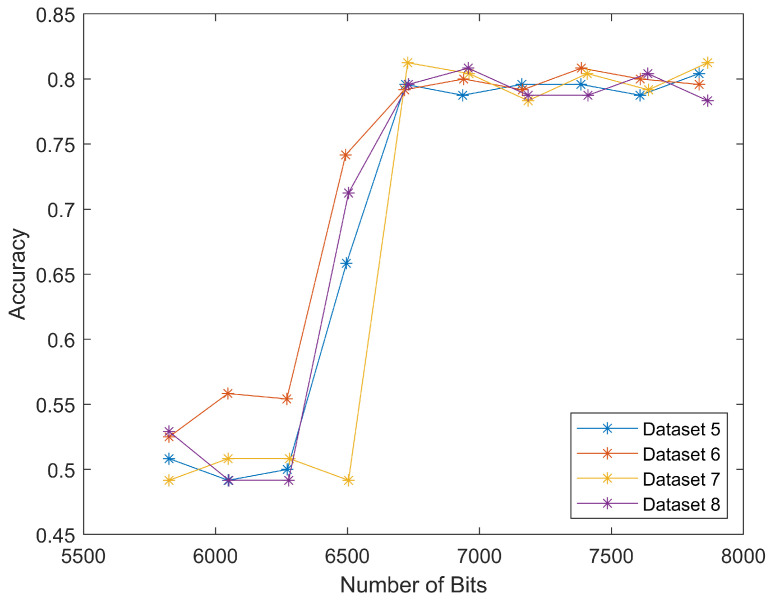
Classification accuracies for datasets five to eight. The similar shape of the four curves proves that the LSTM model can successfully classify compressed domain data.

These two curves matched the trend in Figure 9, because the input data only differed in several segments, from “Start of image” to “Scan header”, as shown in Figure 4. After adding this length back (in our case it was 2624 bits), we put these four curves together, as shown in Figure 11. The well-matched curves again proved that we could only use scan segments to reduce computational load while maintaining relatively high classification accuracies.

The four curves started at the same *x* values, but ended differently, because datasets five and six also discarded the “End of image” marker, as shown on the rightmost in Figure 4. This marker took four hexadecimal values as *FFD9*, which could be translated to 16 bits, and thus make up the difference.

### 4.5. Savings on Storage and Time

In order to better assess how much space and time we could save by using compression domain classifications, we recorded the bits per input and the running time of each training epoch in Table 5. All the experiments were conducted on a computer using Windows 10 with Intel Core i7-7700HQ, which had a clock rate of 2.80 GHz. The MATLAB R2022a was used as the platform and no GPU was used. For datasets with multiple subsets (all datasets except for three and four), only one subset was evaluated for demonstration.

First, we had the original grayscale images of size 50×50 with a bit depth of eight. As LSTM only took sequential input, we reshaped the image to a 1D vector of 2500 pixels, during which the spatial correlation was significantly weakened. These all contribute to the fact that the classification accuracy was only 82.92%, which was lower than using CNN in our previous paper [8]. Regardless of the suboptimal classification accuracy, we could still use this case as the baseline to measure the savings on storage size and computation time if we used compressed byte streams as inputs to classifiers.

For the original image set and datasets one through four, we had to perform decompression first to achieve DCT coefficients. So, we ran the *imread* function on each of the 1600 images. The total decompression time was 1.306 seconds, which was added to the running time of the five mentioned datasets. As image size was 50×50, the total number of MCUs was ${\left(ceil\left(\frac{50}{8}\right)\right)}^{2}=49$.

We could see that all the testing datasets had a large reduction in the input size compared to the original images. Most of the storage saved was translated to a decrease in running time. Note that for datasets six and eight, the running time was even higher than that of the original images. This was caused by the input being binary bitstreams. Though they occupied less storage, when fed into LSTM, the network still treated them as regular values regardless of their bit depth. So, basically, the training time largely depended on the length of the input.

In general, we could infer that the compression domain data would provide similar classification accuracies, and save storage and running time as well. This effect would be more significant if applying the same algorithm to images with larger sizes, or images with high compression ratios.

## 5. Conclusions

The curves shown in previous sections proved that it was possible to classify malaria-infected cells based on partially decoded image segments or even the whole bitstream. The DCT coefficients from datasets one and two could be separated easily, though achieving these values required full decompression. For the DC values after DPCM and VLC in datasets three and four, the drop in accuracy indicated that VLC would destroy the correlation, making LSTM difficult to generalize. Datasets five to eight showed clear evidence for successful classification in the compression domain, even for the reduced size of the input. The good performance could be credited to all images being resized under the same sizes and compressed under MATLAB, so all the header files were the same, including two Huffman tables and the quantization table. These bits contributed no extra information to classification, so we could discard them.

In general, we managed to classify red blood cells in both the pixel domain (DCT coefficients) and compression domain (bitstream). The experimental results showed that we could have partial decompression and obtain similarly good results. We could even feed the neural network with the original bitstream (or bytestream in decimal) of the image and well separated the two classes. The reduced size of the input and skipping the decompression would both lead to saving in computation, as demonstrated by our simulations.

The result could be extended to more general cases for classification in the deep-learning area. Instead of decoding the whole JPEG compressed bitstream to extract the DCT coefficient, or using the original pixel values as input, directly classifying the bitstream might provide similar benefits by reducing the computation time and storage requirement.

## Figures and Tables

**Table 1:** Sample number of Train, test and validation sets.

	Infected	Normal
Training	558	562
Testing	122	118
Validation	120	120

**Table 2:** Extract 12 combinations of coefficients from Table 3. Each set consists of a DC component with a certain number of AC values, which were retrieved either after DCT or after quantization.

	Coefficients after DCT	Coefficients after Quantization
DC	815.88	102
DC + 1AC	815.88, 17.85	102, 3
DC + 2AC	815.88, 17.85, 6.05	102, 3, 1
DC + 3AC	815.88, 17.85, 6.05, −13.82	102, 3, 1, −2
DC + 4AC	815.88, 17.85, 6.05, −13.82, 6.54	102, 3, 1, −2, 1
DC + 5AC	815.88, 17.85, 6.05, −13.82, 6.54, 25.05	102, 3, 1, −2, 1, 5

**Table 3:** Pixels values for a MCU after performing level shift.

122	101	89	94	97	90	89	98
118	109	100	97	99	102	105	106
111	114	108	98	98	107	111	108
108	110	106	99	95	98	102	105
109	102	101	104	99	91	95	108
109	99	99	108	106	94	98	114
106	101	100	104	103	98	100	106
101	104	102	95	93	97	96	91

**Table 4:** All eight datasets from different stages during JPEG compression. Input streams are either decimal numbers or binary numbers.

Number	Content	Form
1	DC values + 0–5 AC values directly after DCT	decimal
2	DC values + 0–5 AC values after quantization	decimal
3	DC values after DPCM	decimal
4	DC values after VLC	binary
5	variable length scan segment without header & marker	decimal
6	variable length scan segment without header & marker	binary
7	variable length whole string	decimal
8	variable length whole string	binary

**Table 5:** Storage and time saved for all datasets. The trade-off can be made between lower accuracies with fewer computational resources (compression domain data), and higher accuracies with more computational resources (original image).

Dataset	Input Size (bits)	Input Size Reduced	Running Time (s)	Time Saved	Accuracy	How Input Size Is Calculated
original	20,000	0	18.118	0	82.92%	50×50×8: image size × bit depth
1	882	95.59%	4.719	73.95%	81.67%	49×18: Number 18 includes 1 bit for sign, 10 bits for number from 0–1023, 7 bits for number after decimal point
2	392	98.04%	6.09	66.39%	85.42%	49×8: Number 8 indicates number of bits for DCT
3	392	98.04%	4.555	74.86%	94.17%	coefficients from −127 to 127
4	184.17	99.08%	5.289	70.81%	69.58%	average of input binary bitstream
5	5208	73.96%	8.249	54.47%	80.42%	651×8: 8: numbers of bits for value from 0–255
6	5208	73.96%	32.03	−76.79%	79.58%	average of input binary bitstream
7	7864	60.68%	10.58	41.61%	81.25%	983×8: 8: numbers of bits for value from 0–255
8	7864	60.68%	46.12	−154.55%	78.33%	average of input binary bitstream

**Figure A1:**
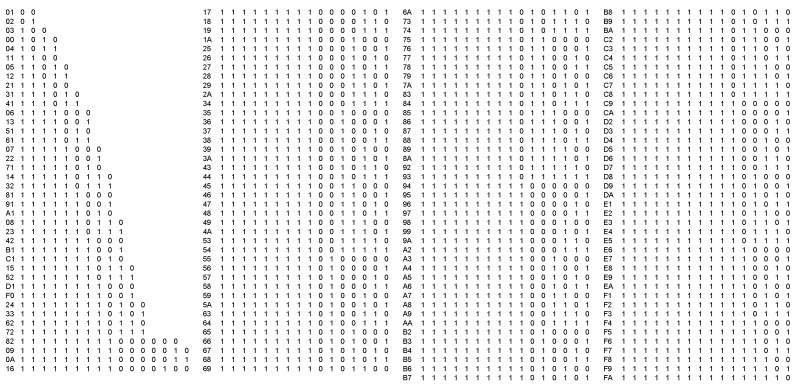
AC Huffman table. The wider range of AC coefficients requires more code-words.

## Data Availability

Available online: https://peir-vm.path.uab.edu/wsi.php?slide=IPLab11Malaria, accessed on 15 March 2022.

## Abbreviations

The following abbreviations are used in this manuscript:

JPEG	Joint Photographic Experts Group
MCU	minimum coded unit
DCT	Discrete cosine transform
DPCM	Differential pulse-code modulation
VLC	Variable length coding
LSTM	Long short-term memory
WSI	Whole slide image
DFT	Discrete Fourier transform
SVM	Support vector machine
CNN	Convolutional neural network

## Appendix A

This appendix includes tables and matrices used in the JPEG compression of MATLAB. For the DC Huffman table in Table A1 and AC Huffman table in Figure A1, the pair of values are hexadecimal values for the letters, and the zeros and ones are the corresponding codewords.

For the quantization table in Table A2, the left top value 8 is the lowest frequency and the right bottom value 50 is the highest frequency, which matches the DCT coefficients.

**Table A1:** DC Huffman table. Less frequent symbols are assigned longer code-words.

Symbols	Code-Words								
00	0	0							
01	0	1	0						
02	0	1	1						
03	1	0	0						
04	1	0	1						
05	1	1	0						
06	1	1	1	0					
07	1	1	1	1	0				
08	1	1	1	1	1	0			
09	1	1	1	1	1	1	0		
0A	1	1	1	1	1	1	1	0	
0B	1	1	1	1	1	1	1	1	0

**Table A2:** Quantization table. The corresponding JPEG quality factor is 75%.

8	6	5	8	12	20	26	31
6	6	7	10	13	29	30	28
7	7	8	12	20	29	35	28
7	9	11	15	26	44	40	31
9	11	19	28	34	55	52	39
12	18	28	32	41	52	57	46
25	32	39	44	52	61	60	51
36	46	48	49	56	50	52	50

Zig-zag order as shown in Table A3 determines the order when flattening the coefficient matrix to a vector.

**Table A3:** Zig-zag order. Values in an MCU will be translated to a vector according to this order.

0	1	5	6	14	15	27	28
2	4	7	13	16	26	29	42
3	8	12	17	25	30	41	43
9	11	18	24	31	40	44	53
10	19	23	32	39	45	52	54
20	22	33	38	46	51	55	60
21	34	37	47	50	56	59	61
35	36	48	49	57	58	62	63
